# Takotsubo cardiomyopathy in the setting of severe hyponatremia and beer potomania: A case report

**DOI:** 10.1002/ccr3.6717

**Published:** 2022-12-09

**Authors:** Sheriff N. Dodoo, Alicia Agyemang‐Sarpong, Nchang Taka, Richmond A. Akatue, Marcus L. Williams

**Affiliations:** ^1^ Department of Hospital Medicine Piedmont Newnan Hospital Newnan Georgia USA; ^2^ Department of Cardiology Northeast Georgia Medical Center‐Georgia Heart Institute Gainsville Georgia USA; ^3^ Postgraduate Medical Education Harvard Medical School Boston Massachusetts USA; ^4^ Department of Cardiology WellStar West Georgia Medical Center LaGrange Georgia USA; ^5^ Department of Internal Medicine Meharry Medical College Nashville Tennessee USA; ^6^ Department of Cardiology Emory University at LaGrange LaGrange Georgia USA

**Keywords:** apical ballooning, beer potomania, echocardiogram, electrocardiogram, hyponatremia, left heart catherization, status epilepticus, takotsubo cardiomyopathy

## Abstract

Takotsubo cardiomyopathy (TC), an acute cardiac event is often associated with acute emotional stress, usually in the setting of cardiovascular risk factors. This case report attempts to review one of the triggers of TC beer potomania‐induce hyponatremia with imaging findings that shows the link between severe hyponatremia and TC.

## Graphical abstract

Severe hyponatremia as a trigger and potential etiology of Takotsubo cardiomyopathy is poorly understood and under‐recognized. Our case emphasizes the need to consider Takotsubo cardiomyopathy as a potential differential diagnosis in patients presenting with severe hyponatremia from beer potomania in the setting of acute coronary syndrome without significant coronary artery disease.

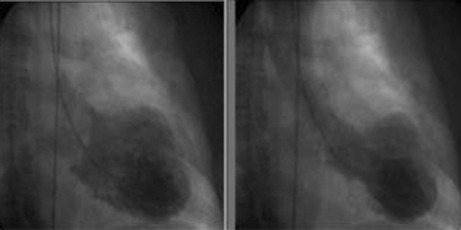

## 1 BACKGROUND

Takotsubo cardiomyopathy (TC), first described in Japan in 1990, is an acute cardiac condition that involves transient systolic dysfunction due to ballooning of the apex and/or mid segments of the left ventricle.^1^ TC is also known as “apical ballooning syndrome,” “stress‐induced cardiomyopathy,” “broken heart syndrome,” and “ampulla cardiomyopathy”. The name Takotsubo was derived from the Japanese word for octopus emblematic of the appearance of the left ventricle on ventriculography during an acute attack. The typical TC includes apical ballooning during systole due to hypokinesis or akinesis of the apex or mid ventricle and hyperkinesis of the basal walls. Atypical variants of TC include hypokinesis of the mid‐ventricle alone,^2^ hypokinesis of the base, and global hypokinesis.^3^

Takotsubo cardiomyopathy patients are typically seen in postmenopausal Asian or Caucasian women. Gianni et al reported that 88.8% of 286 reported TC patients were women. The mean age ranges from 61 to 76 years.^4^ The exact prevalence of TC is unknown, but researchers have reported that 1.7–2.2% of suspected ACS patients have TC.^5^, ^6^, ^7^ TC is usually but not always brought on by an acute medical illness or an intense mental or physical stressor.^8^ TC patients typically present with symptoms similar to ACS, including chest pain with echocardiographic changes and elevated cardiac markers. However, upon angiography, no significant coronary artery obstruction is appreciated. Sadamatsu et al reported two cases with apical wall abnormalities and reduced coronary flow without coronary stenosis.^9^

We report a case of Takotsubo cardiomyopathy in a patient who initially presented with severe hyponatremia from beer potomania. This patient did not present with chest pain; however, the apical ballooning and negative coronary artery disease were discovered on left heart catherization and ventriculogram.

## 2 CASE PRESENTATION

A 56‐year‐old African American male with medical history significant for hypertension, hyperlipidemia, and alcohol dependence who presented with incoherent speech with altered mentation. He reported dyspnea with mild exertion. He denied chest pain, orthopnea, paroxysmal nocturnal dyspnea, or pedal swelling. He has been binge drinking several cans of beer, about 24 of 24 ‐Oz can, prior to presentation. This was, following, a sudden incarceration and imprisonment of his wife. Patient had his last drink 5 h prior to presentation to the emergency room.

Examination revealed a disheveled middle‐age African American male who was confused and inebriated. His Vital signs revealed blood pressure 129/67 mmHg, pulse 73beats/min, and body temperature 99.4 F. He was somnolent but easily arousable and oriented to person and place but not to time or situation. Neurologic examination showed no focal neurological deficits. The rest of his physical examination yielded no addition findings.

**FIGURE 1:**
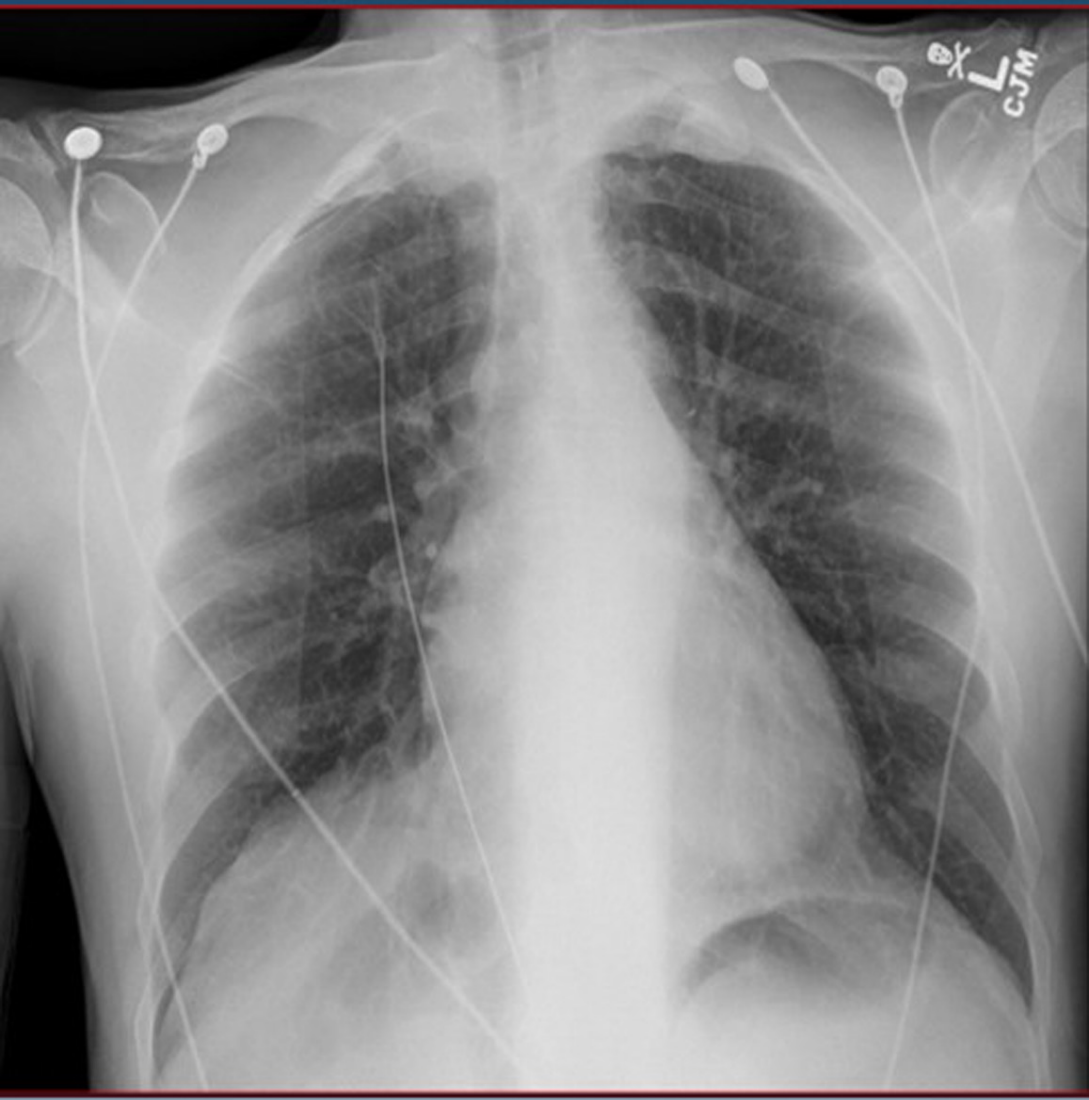
Initial Electrocardiogram showing normal sinus rhythm

**FIGURE 2:**
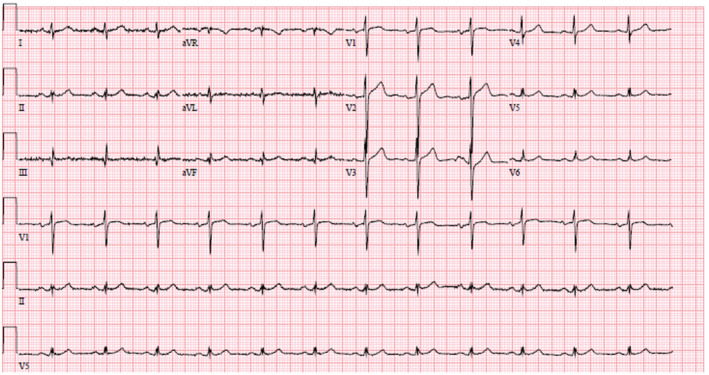
Initial Chest x‐ray showing no acute cardiopulmonary process

Laboratory investigations including biochemical and hematologic results obtained in the ER are listed below (Table 1). This revealed serum sodium 102 mmol/L, serum osmolality 245 mOsm/L, urine osmolality 44 mOsm/L, urine sodium 7 mmol/L, blood alcohol level 221 mg/dL, and creatine kinase 7810 units/L. Random urine drug screen was positive for opiates. Initial electrocardiogram showed normal sinus rhythm (Figure 1). Chest X‐ray showed no acute cardiopulmonary process (Figure 2). About 45 min after presentation at the emergency department, he experienced violent incessant episodes of generalized clonic–tonic seizure episode involving all limbs. This was concerning for status epilepticus and required sedation with phenobarbital and intubation for airway protection at the medical intensive care unit at our community hospital. Nephrology, critical care, and neurology consultation were subsequently placed.

**TABLE 1:** Biochemical and hematologic laboratory results at the time of initial presentation in the emergency room

Test	Result	Reference
White blood cells (WBC)	7.2 × 10^3^ μl	3.4–10.8 × 10^3^ μl
Hemoglobin (Hb)	13.9 g/dl	12.6–17.7 g/dl
Hematocrit (Hct)	35.80%	37.5–51.0%
Platelet count	253 × 10^3^ μl	150–379 × 10^3^ μl
Serum sodium (Na)	102 mmol/L	134–144 mmol/L
Serum potassium (K)	4.2 mmol/L	3.5–5.2 mmol/L
Serum chloride (Cl)	73 mmol/L	96–106 mmol/L
Serum bicarbonate	22 mmol/L	18–29 mmol/L
Blood urea nitrogen (BUN)	6.0 mg/dl	6.0–24 mg/dl
Creatinine	0.3 mg/dl	0.6–1.2 mg/dl
Serum glucose	103 mg/dl	65–100 mg/dl
Serum calcium	9.1 mg/dl	8.7–10.2 mg/dl
Serum phosphate	3.1 mg/dl	2.5–4.5 mg/dl
Serum magnesium	2.1 mg/dl	1.7–2.2 mg/dl
Aspartate aminotransferase (AST)	42 IU/L	0.0–40 IU/L
Alanine aminotransferase (ALT)	34 IU/L	0.0–44 IU/L
Alkaline phosphatase (ALP)	101 IU/L	39–117 IU/L
Total bilirubin	0.4 mg/dl	0.0–1.2 mg/dl
Direct bilirubin	0.1 mg/dl	0.0–0.3 mg/dl
Total protein	7.8 g/dl	6–8.3 g/dl
Albumin	3.0 g/dl	3.5–5.5 g/dl
International normalized ratio (INR)	1	≤1.1
Serum uric acid	2.8 mg/dl	3.4–7.0 mg/dl
Thyroid‐stimulating hormone (TSH)	1.25 μIU/ml	0.45–4.5 μIU/ml
NT‐proB‐type Natriuretic peptide (BNP)	125 pg/ml	0.00–900.00 pg/ml
Troponin	0.024 ng/ml	0.000–0.034 ng/ml
Blood ethanol	221 mg/dl	<10 mg/dl
Creatine kinase	7810 units/L	55–170 units/L
Serum osmolarity	245 mOsm/L	275–295 mOsm/kg
Urine osmolarity	44 mOsm/L	50–1200 mOsm/kg
Random urine sodium	7 mmol/L	20–40 mmol/L
Ketones in urine	Trace	Absent

Patient was given hypertonic saline with close monitoring of his serum sodium and electrolytes. The rise in serum sodium was 0.5‐1 mmol/L/h, and serum sodium gradually improved to 120 over 2 days. The patient's chest X‐ray demonstrated possible right middle lobe pneumonia, and he was started on broad‐spectrum antibiotics of ceftriaxone and azithromycin intravenously. The patient continued to be on mechanical ventilation and multiple attempts at extubating failed.

**FIGURE 3:**
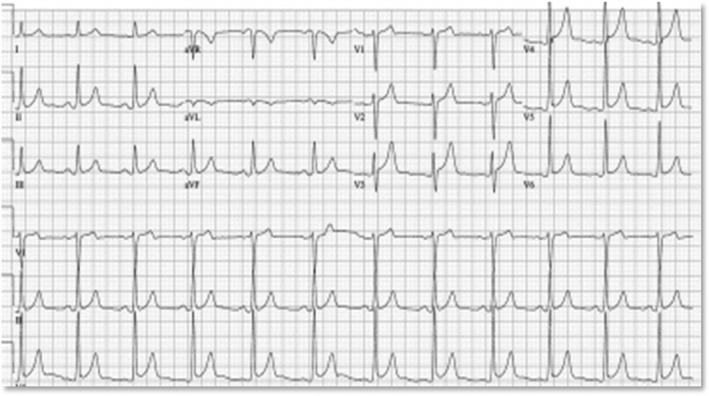
EKG showing early repolarisation abnormality in left lateral leads

Over the next 24–48 h, a change was noted on telemetry monitoring concerning for ST elevation and a 12‐lead electrocardiogram showed early repolarization abnormalities in the left lateral leads (Figure 3). Follow‐up cardiac enzymes done showed troponin of 4.30 mg/ml, creatine kinase‐ MB 50 U/L, and creatine kinase 1293 U/L.” The ST elevations did not qualify for classification as STEMI; however, he required urgent treatment for NSTE‐ACS. The patient was, subsequently transferred to a neighboring hospital with percutaneous coronary intervention and cardiac catheterization capability.

He stayed on mechanical ventilation several days. Echocardiogram done prior to the left heart catherization showed left ventricular ejection fraction of 30% with severe mid‐distal and apical hypokinesis and ballooning, and relaxation abnormality of left ventricular hypertrophy with mild concentric left ventricular hypertrophy was also appreciated (Video S1). The patient received aspirin, metoprolol, and lisinopril orally with heparin intravenously as medical therapy.

**FIGURE 4:**
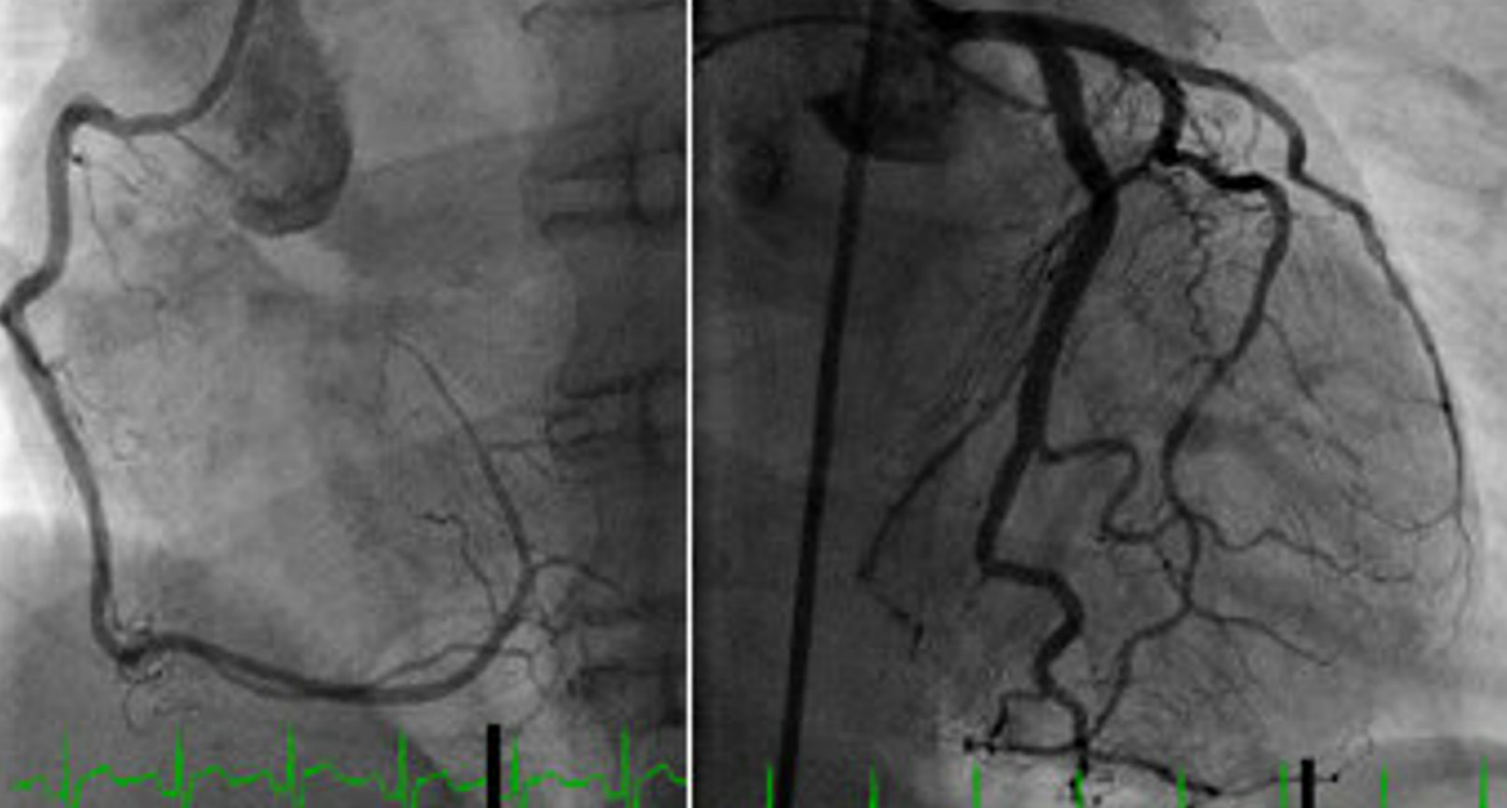
Heart catheterization showing left and right coronaries without stenosis

**FIGURE 5:**
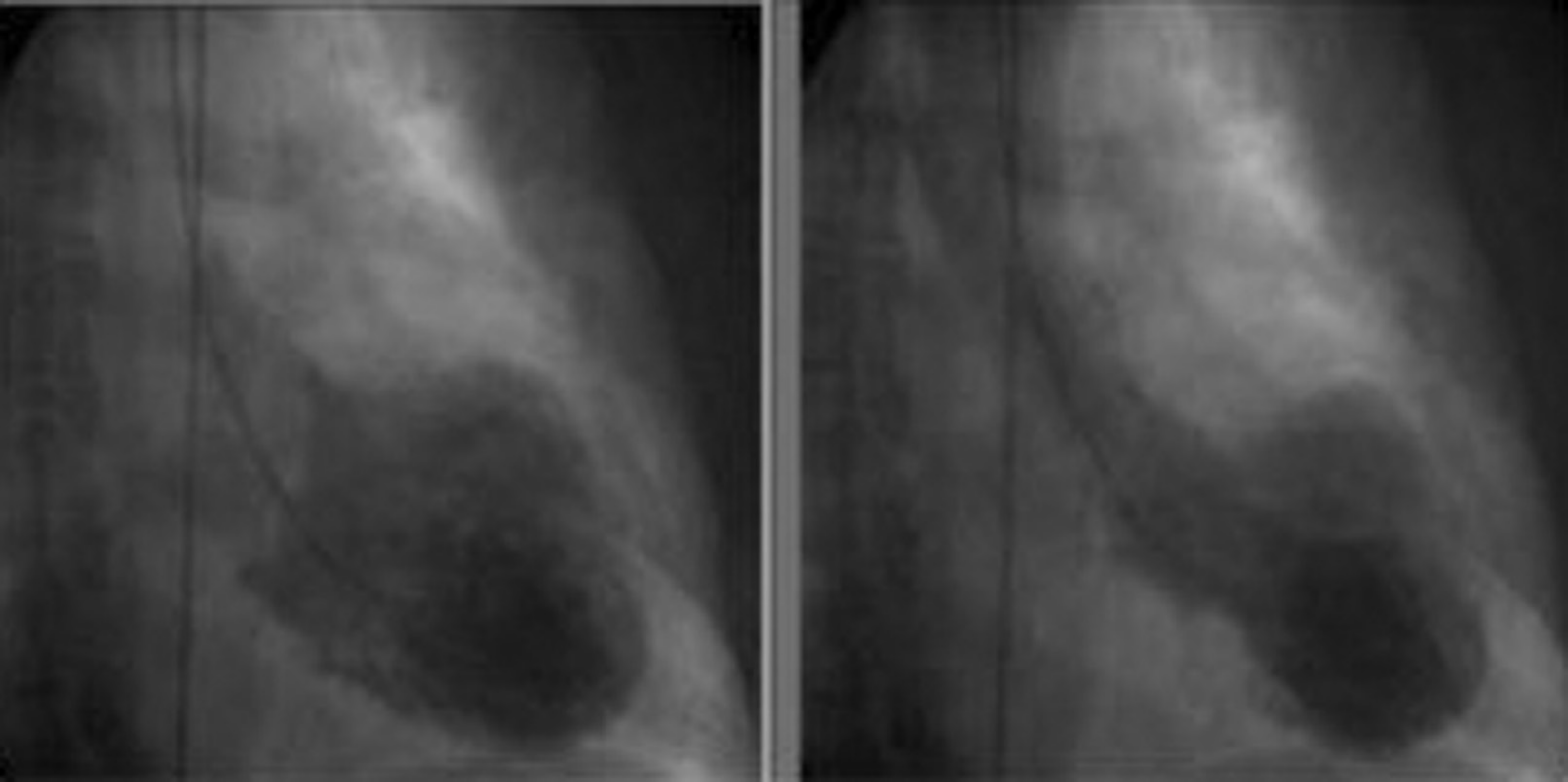
Ventriculogram imaging showing the apical and mid segment left ventricular. Akinesis and ballooning, taking the shape of the proverbial Japanese octopus, Takotsubo cardiomyopathy. Left image: apical left ventricular Akinesis and ballooning. Right image: Mid‐segment left ventricular ballooning

The left heart catheterization (LHC) done showed no evidence of obstructive CAD (Figure 4). There was no evidence of coronary vasospasm. LV angiogram showed apical ballooning and hypokinesis of anteroseptal left ventricle concerning for Takotsubo cardiomyopathy (Figure 5). The patient was monitored closely after the LHC. Troponin peaked at 33.0 mg/ml and subsequently trended down 0.04 mg/ml, 3 days after the LHC.

He remained on hypertonic saline with increases of his serum sodium to 123 mmol/L. The hypertonic saline was stopped when his serum sodium increased to 129 mmol/L. The sodium remained stable at 128–130 mmol/L. He was successfully extubated, after 4 days of mechanical ventilation. His mental status slowly improved and began to respond to commands. Patient made steady improvement in his clinical condition, antibiotics was discontinued and was discharge after 6 days of hospital stay.

An echocardiogram done a month post admission during a follow‐up clinic visit to our hospital showed left ventricular ejection fraction of 55% with resolution of apical hypokinesis and ballooning (Video S2).

## 3 DISCUSSION

The patient's initial presentation of a low serum sodium of 101 mmol/L raised the possibility of number of differential diagnoses including syndrome of inappropriate anti diuretic hormone (SIADH), dehydration, congestive heart failure, chronic kidney disease, cerebral wasting syndrome, psychogenic polydipsia, and beer potomania. Low urine osmolarity and low urine sodium levels excluded dehydration, SIADH, and cerebral wasting syndrome as the cause of this patient's hyponatremia.^10^ The patient's denial of drinking excessive water also ruled out psychogenic polydipsia. This patient's noncontributory initial physical examination, along with chest X‐ray, without any acute intrathoracic process with a normal BNP and renal function essentially ruled out congestive heart failure and renal insufficiency.

His history of alcohol abuse, including clinical presentation of lethargy and disheveled appearance, along with his laboratory work up of low serum osmolarity, urine osmolarity, and low urine sodium results and absence of possible explanation, led us to the possibility of beer potomania accounting for the patient's hyponatremia. Our eventual working diagnosis of the patient's hyponatremia was likely related to alcohol, and hence, the patient possibly had beer potomania evidenced by low urine sodium and severe hyponatremia. This together led to the status epilepticus our patient experienced. We hypothesize that his severe hyponatremia may have cause the Takotsubo cardiomyopathy (TC), especially in the context of epileptic seizures. The TC evidenced by the absence of coronary artery stenosis on LHC and presence of apical ballooning on ventriculogram and echocardiogram. This was buttressed by a low left ventricular ejection fraction of 30% and its eventual improvement to 55% over a relatively short period of time of a month.

The pathogenesis of TC is not fully understood, but the proposed mechanisms include endogenous catecholamine excess, multivessel coronary artery vasospasm, and microvascular dysfunction. The most favored mechanism is endogenous catecholamine excess leading to microvascular spasm or dysfunction resulting in myocardial stunning.^11^ Others have also discussed a direct toxicity of cardiomyocyte from the large amount of circulating catecholamines.^12^ In support of the endogenous catecholamine excess hypothesis, a mouse model showed that a high level of epinephrine had negatively inotropic effect on cardiomyocytes due to a switch from beta‐2 adrenoceptor Gs protein signaling to Gi protein signaling. It is speculated that the effect is greatest on the apex of the myocardium because of a higher density of beta‐2 adrenoceptors.^13^ Additionally, Ellison et al found that high doses of isoproterenol cause diffuse death of myocytes while sparing cardiac stem cells in rats allowing for rapid recovery of the myocardium.^14^

Akashi et al in another study reported that TC patients had an increased myocardial ^123^I‐metaodobenzlguanide (^123^I‐MBG) washout rate which indicates an increased norepinephrine release from sympathetic nerve endings or increased clearance of ^123^I‐MBG by extra neural tissues. Ultimately, the increased wash out rate correlated to increased plasma norepinephrine levels in TC patients.^15^

There are a few case reports of TC in the setting of moderate to severe hyponatremia described in the body of literature.^16^, ^17^, ^18^, ^19^, ^20^, ^21^ Hyponatremia has not been thought to be linked to Takotsubo cardiomyopathy but perhaps may have an indirect causal relationship. The prevailing theory of this indirect causal relationship is a stress‐induced catecholamine storm causing a direct toxic effect on the myocardium or indirect effect by coronary vasculature constriction. The mechanistic connection is still not clear; however, it has been suggested that hyponatremia could interfere with myocardial inotropy by modifying the cardiomyocytic sodium‐calcium exchange pump resulting in myocardial swelling associated with hypotonicity.^16^

Indeed, transient positive inotropic effects on the myocardium were observed in rat hearts, and the degree of positive inotropy correlated with the degree of hyponatremia.^22^ There have been cases reported of Takotsubo cardiomyopathy in the setting of “isolated hyponatremia,” and it has been suggested that in post‐menopausal women presenting with acute coronary syndrome‐like symptoms and hyponatremia, Takotsubo cardiomyopathy should be considered within the differential diagnoses.^17^ Takotsubo cardiomyopathy arising as a direct consequence of hyponatremia is an unexplored mechanism for this poorly understood disease process. The prevailing theories for the pathogenesis of TC involve excessive catecholamine action on the myocardium causing stunning either directly or through ischemia by causing multivessel epicardial or microvascular spasm.^23^

There has also been a long‐recognized connection between TC and stress, particularly strong emotional stress, which suggests that there may be a neurohumoral connection that precipitates TC. Interestingly, TC has been found in cases of subarachnoid hemorrhage and stroke, and neurologists have advanced the idea “neurogenic stunning” to describe this reversible cardiomyopathy in the setting of brain injury in the absence of coronary artery disease^23^ Norepinephrine release in the myocardium is increased as a result of hypothalamic ischemia from a subarachnoid hemorrhage and may be the cause of the myocardial injury observed.^24^ Furthermore, this neurogenic stunning effect is dampened when there is a disruption of neural innervation of the myocardium as in diabetes or heart transplant.^25^ Neurocardiac lesions also occur in adrenalectomized animals, but to a lesser extent, further strengthening the neurogenic stunning theory.^26^

Although ischemia as a direct cause of TC is still being debated, the dysfunctional myocardium in TC follows a neural rather than vascular distribution, as there is a much higher concentration of adrenergic receptors in areas around myocardial arterioles than in areas adjacent to epicardial coronary arteries.^27^

## 4 CONCLUSION

Takotsubo cardiomyopathy's preferential effects on the sub‐endocardial myocytes manifest as an increased propensity for arrhythmia; this combined with excessive catecholamines, which can induce arrhythmia even in healthy myocardium, may be a major cause of sudden death in neurologic disease including subarachnoid hemorrhage, stroke, head trauma, and increased intracranial pressure.

With the wealth of evidence connecting the brain's effect on the heart, perhaps hyponatremia indirectly causes TC by first causing cerebral edema. The resulting neurological disturbance results in excessive catecholamine action on the myocardium which manifests as TC.

## AUTHOR CONTRIBUTIONS

**Sheriff Dodoo:** Conceptualization; data curation; formal analysis; investigation; methodology; project administration; supervision; writing – original draft; writing – review and editing. **Alicia Agyemang‐Sarpong:** Data curation; writing – original draft; writing – review and editing. **Taka Nchang:** Writing – review and editing. **Richmond Akatue:** Writing – review and editing. **Marcus L Williams:** Writing – review and editing.

## FUNDING INFORMATION

No funding was secured for this study.

## CONFLICT OF INTEREST

The authors declare that they have no competing interest to disclose.

## CONSENT

Written informed consent was obtained from the patient for publication of this case report and any accompanying images. A copy of the written consent is available for review by the Editor‐in‐Chief of this journal.

## Supporting information


Video S1

Video S2

## Data Availability

Available on demand.

## References

[1] Sato H , Tateishi H , Uchida T , et al. Takotsubo type cardiomyopathy due to multivessel spasm. In: Kodama K , Haze K , Hon M , eds. Clinical Aspect of Myocardial Injury: from Ischemia to Heart Failure. Kagaku Hyoronsha; 1990:56‐64.

[2] Eitel I , von Knobelsdorff‐Brenkenhoff F , Bernhardt P , et al. Clinical characteristics and cardiovascular magnetic resonance findings in stress (takotsubo) cardiomyopathy. JAMA. 2011;306(3):277‐286. doi:10.1001/jama.2011.992 21771988

[3] Win CM , Pathak A , Guglin M . Not takotsubo: a different form of stress‐induced cardiomyopathy—a case series. Congest Heart Fail. 2011;17(1):38‐41. 21272226 10.1111/j.1751-7133.2010.00195.x

[4] Akashi YJ , Barbaro G , Sakurai T , et al. Cardiac autonomic imbalance in patients with reversible ventricular dysfunction takotsubo cardiomyopathy. Q J Med. 2007;100:335‐343. 10.1093/qjmed/hcm028 17483198

[5] Bybee KA , Prasad A , Barsness GW , et al. Clinical characteristics and thrombolysis in myocardial infarction frame counts in women with transient left ventricular apical ballooning syndrome. Am J Cardiol. 2004;94:343‐346. 15276100 10.1016/j.amjcard.2004.04.030

[6] Akashi YJ , Goldstein DS , Barbaro G , Ueyama T . Takotsubo cardiomyopathy: a new form of acute, reversible heart failure. Circulation. 2008;118(25):2754‐2762. 19106400 10.1161/CIRCULATIONAHA.108.767012 PMC4893309

[7] Matsuoka K , Okubo S , Fujii E , et al. Evaluation of the Arrhythmogenecity of stress‐induced “takotsubo cardiomyopathy” from the time course of the 12‐Lead surface electrocardiogram. Am J Cardiol. 2003;92:230‐233. 12860233 10.1016/s0002-9149(03)00547-2

[8] Gianni M , Dentali F , Grandi AM , Sumner G , Hiralal R , Lonn E . Apical ballooning syndrome or takotsubo cardiomyopathy: a systematic review. Eur Heart J. 2006;27:1523‐1529. 16720686 10.1093/eurheartj/ehl032

[9] Sadamatsu K , Tashiro H , Maehira N , Yamamoto K . Coronary microvascular abnormality in the reversible systolic dysfunction observed after noncardiac disease. Jpn Circ J. 2000;64:789‐792. 11059622 10.1253/jcj.64.789

[10] Nakajima H , Okada H , Hirose K , et al. Cerebral salt‐wasting syndrome and inappropriate antidiuretic hormone syndrome after subarachnoid hemorrhaging. Intern Med. 2017;56(6):677‐680. 28321069 10.2169/internalmedicine.56.6843 PMC5410479

[11] Dote K , Sato H , Tateishi H , et al. Myocardial stunning due to simultaneous multivessel coronary spasms: a review of 5 cases. J Cardiol. 1991;21(2):203‐214. 1841907

[12] Nef HM , Möllmann H , Kostin S , et al. Tako‐tsubo cardiomyopathy: intraindividual structural analysis in the acute phase and after functional recovery. Eur Heart J. 2007;28(20):2456‐2464. doi:10.1093/eurheartj/ehl570 17395683

[13] Lyon AR , Rees PS , Prasad S , et al. Stress (takotsubo) cardiomyopathy‐‐a novel pathophysiological hypothesis to explain catecholamine‐induced acute myocardial stunning. Nat Clin Pract Cardiovasc Med. 2008;5(1):22‐29. doi:10.1038/ncpcardio1066 18094670

[14] Ellison GM , Torella D , Karakikes I , et al. Acute ß‐adrenergic overload produces myocyte damage through calcium leakage from ryanodine Receptor‐2 but spares cardiac stem cells. J Biol Chem. 2007;282:11397‐11409. 17237229 10.1074/jbc.M607391200 PMC2276680

[15] Akashi YJ , Nakazawa K , Sakakibara M , Miyake F , Musha H , Sasaka K . 123I‐MIBG myocardial scintigraphy in patients with “takotsubo” cardiomyopathy. J Nucl Med. 2004;45:1121‐1127. 15235057

[16] Santos M , Dias V , Meireles A , et al. Hyponatremia – an unusual trigger of takotsubo cardiomyopathy. Rev Port Cardiol (English Edition). 2011;30(11):845‐848. 10.1016/j.repc.2011.09.006 22030327

[17] Abouezzeddine O , Prasad A . Apical ballooning syndrome precipitated by hyponatremia. Int J Cardiol. 2010;145(1):E26‐E29. 19195723 10.1016/j.ijcard.2008.12.195

[18] Rivera JM , Locketz AJ , Fritz KD , et al. "broken heart syndrome" after separation (from OxyContin). Mayo Clin Proc. 2006;81(6):825‐828. doi:10.4065/81.6.825 16770984

[19] Dib C , Asirvatham S , Elesber A , Rihal C , Friedman P , Prasad A . Clinical correlates and prognostic significance of electrocardiographic abnormalities in apical ballooning syndrome (takotsubo/stress‐induced cardiomyopathy). Am Heart J. 2009;157(5):933‐938. doi:10.1016/j.ahj.2008.12.023 19376324

[20] Cocco G , Chu D . Stress‐induced cardiomyopathy: a review. Eur J Intern Med. 2007;18(5):369‐379. doi:10.1016/j.ejim.2007.02.021 17693225

[21] Patel HM , Kantharia BK , Morris DL , Yazdanfar S . Takotsubo syndrome in African‐American women with atypical presentations: a single‐center experience. Clin Cardiol. 2007;30(1):14‐18. doi:10.1002/clc.21 17262772 PMC6653722

[22] Kolar F , Cole WC , Ostadal B , et al. Transient inotropic effects of low extracellular sodium in perfused rat heart. American journal of physiology – heart and circulatory. Phys Ther. 2015;259(3):H712‐H719. 10.1152/ajpheart.1990.259.3.H712 2396684

[23] Prasad A , Lerman A , Rihal C . Apical ballooning syndrome (Tako‐tsubo or stress cardiomyopathy): a mimic of acute myocardial infarction. Am Heart J. 2008;155(3):408‐417. 18294473 10.1016/j.ahj.2007.11.008

[24] Kono T , Morita H , Kuroiwa T , Onaka H , Takatsuka H , Fujiwara A . Left ventricular wall motion abnormalities in patients with subarachnoid hemorrhage: neurogenic stunned myocardium. J Am Coll Cardiol. 1994;24(3):636‐640. doi:10.1016/0735-1097(94)90008-6 8077532

[25] Samuels MA . The brain‐heart connection. Circulation. 2007;116(1):77‐84. doi:10.1161/CIRCULATIONAHA.106.678995 17606855

[26] Hawkins WE , Clower BR . Myocardial damage after head trauma and simulated intracranial hemorrhage in mice: the role of the autonomic nervous system. Cardiovasc Res. 1971;5:524‐529. 5160456 10.1093/cvr/5.4.524

[27] Murphree S , Saffitz J . Quantitative autoradiographic delineation of the distribution of Beta‐adrenergic receptors in canine and feline left ventricular myocardium. Circ Res. 1987;60:568‐579. 3036392 10.1161/01.res.60.4.568

